# A New Design of an MOEMS Gyroscope Based on a WGM Microdisk Resonator

**DOI:** 10.3390/s19122798

**Published:** 2019-06-21

**Authors:** Dunzhu Xia, Lingchao Huang, Liye Zhao

**Affiliations:** Key Laboratory of Micro-Inertial Instrument and Advanced Navigation Technology, Ministry of Education, School of Instrument Science and Engineering, Southeast University, Nanjing 210096, China; 220162744@seu.edu.cn (L.H.); liyezhao@seu.edu.cn (L.Z.)

**Keywords:** disk resonator, MOEMS gyroscope, whispering-gallery mode (WGM)

## Abstract

In this paper, we present a new design for a micro-opto-electro-mechanical (MOEMS) gyroscope based on a whispering-gallery mode (WGM) microdisk resonator and MEMS resonator. The mechanical characteristics, frequency split, and quality factor (Q) of the MEMS resonator; the optical characteristics, Q value, and coupling regimes of the WGM resonator; and the coupling between the two resonators were analyzed. Its operation principle—the transformation process from angular velocity to the resonance wavelength of the WGM resonator—is presented at same time. Next, the analysis conclusions were validated with the help of simulations in ANSYS and FDTD (Finite-Difference Time-Domain) Solutions. Afterwards, some key specifications were estimated based on the results of simulations. Lastly, the fabrication process is detailed.

## 1. Introduction

Gyroscopes are essential for a variety of applications ranging from inertial navigation to motion sensing in video games. The typical gyroscope can be realized using capacitive, piezoelectric, electric resistance, or optical methods. In the last few decades, micro-electro-mechanical (MEMS) gyroscopes have been widely used due to their advantages in both volume and the cost of their manufacturing. However, the drawbacks to using an MEMS gyroscope using the capacitive method, which is the main type currently in the market, include suboptimal precision and anti-electromagnetic interference. Thus, they are not suitable for use in military weapons and commercial production, which demand the use of a low-cost gyroscope with high precision. Meanwhile, gyroscopes using optical detection methods do not suffer from the same disadvantages as the MEMS gyroscope and provide superior displacement resolution [1], resilience to electromagnetic interference, and a long-range readout [2]. Furthermore, trends toward the integration of micro-photonics and microelectronics in a silicon-on-insulator (SOI) platform have made a lot of progress during recent years [3]; this makes the micro-opto-electro-mechanical (MOEMS) gyroscope an attractive option.

There are many different kinds of MOEMS inertial sensors according to their micro-photonic components, including grating, an optical cavity, and a wave guide. Among the different micro-photonic components, microdisk and microring resonators have been actively pursued for chip-scale silicon photonics. The high quality factors (Q) and small mode volumes that have been achieved in micrometer-scale devices result in a very long photon life time and strong electric field enhancement inside these resonators. The unique properties of these microdisk resonators make them ideal candidates for many applications including the filtering, sensing, delay, and enhancement of light–matter interactions [4]. Moreover, since the microdisk is a traveling-wave resonator, it is possible to realize a very high transmission efficiency of energy by appropriately designing the waveguide–resonator coupling [5]. Hence, microring and microdisk resonators on different substrates have been used previously as displacement sensors, biosensors, ultrasound sensors, and chemical sensors and in optomechanical systems [6,7,8,9,10,11,12,13]. Therefore, we propose a new type of gyroscope based on the use of whispering-gallery mode (WGM) resonators fabricated on an SOI wafer; the WGM resonator was chosen for the kernel sensing components of the gyroscope. 

The gyroscope presented in this paper can be divided into two parts: the MEMS disk resonator and the microdisk optical resonator. The sense mode of the MEMS disk resonator, when operated in the drive mode under the input signal from the electrodes, will be activated when it is rotating. The WGM resonator was used to measure the deformation of the MEMS disk resonator in the sense mode, which is related to the angular velocity. By measuring the shift of the transmission spectrum of the WGM resonator, the angular velocity could be determined.

## 2. Structure of the MOEMS Gyroscope and Operational Principle of the MEMS Disk Resonator 

### 2.1. Structure of the New MOEMS Gyroscope

In this paper, we designed a new type of MOEMS gyroscope based on both mechanical and optical resonators. 

**Figure 1:**
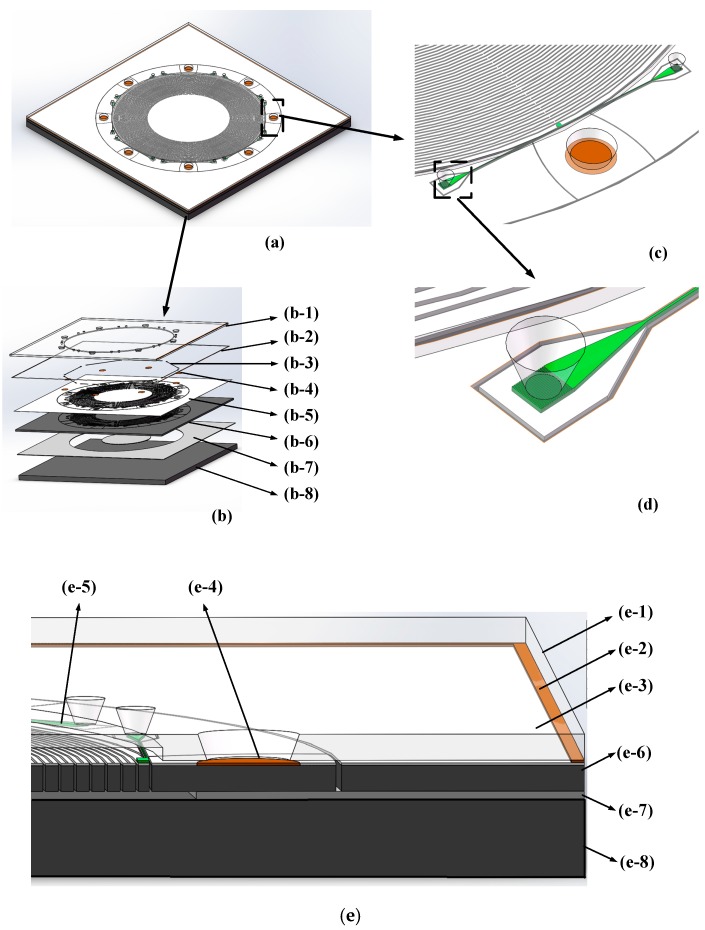
Diagrams of the structure of the micro-opto-electro-mechanical (MOEMS) gyroscope: (**a**) Diagram of the entire gyroscope; (**b**) Exploded view; (**c**,**d**) Partial enlarged drawing; (**e**) Cutaway drawing.

The entire structure of the gyroscope is shown in Figure 1a. The gyroscope was fabricated in an SOI wafer; its components are shown in Figure 1b. Figure 1 (b-1) to (b-8) shows the glass gap, the metal sealed wall, the WGM resonator, the metal pad used for electric signal transmission, the SiO_2_ layer, the MEMS disk resonator, the buried oxide layer, and the handle layer, respectively. Figure 1c,d shows the partial enlarged drawing, in which (c) shows the optical parts of the WGM resonator including the grating couplers, tapers, waveguide, and disk resonant cavities, and (d) shows a more detailed depiction of these optical components.

Figure 1e is a cutaway drawing of the gyroscopes; information about the parts can be found in Table 1. The operational principle and fabrication process are described in the later chapters. 

### 2.2. Operational Principle of the MEMS Disk Resonator

**Figure 2:**
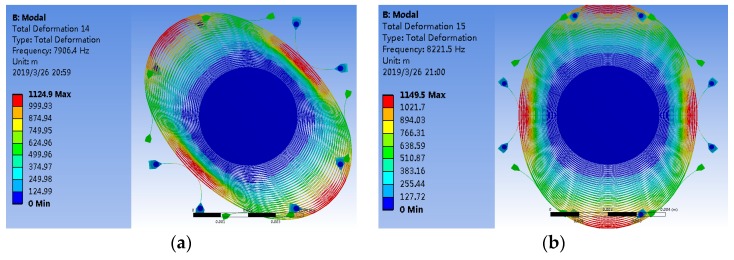
Diagram of the micro-electro-mechanical (MEMS) disk resonator modes: (**a**) Drive mode; (**b**) Sense mode.

The structure of the MEMS disk resonator is shown in Figure 1. From this figure, it can be seen that the resonator consists of two glass substrates with electrical connection lines and bonding pads, an electrode, a disk resonator, and 16 discrete pillar electrodes that encircle the disk and are evenly distributed. Meanwhile, there are 16 cone-shaped feed-through holes located on the top glass substrate patterned with 16 electrodes and a through hole located on the bottom glass for the DC bias connection. The operational principle of the disk resonator is shown in Figure 2; it is driven into oscillation along the drive axis, and then the Coriolis force caused by the rotation in the detecting axis, which is vertical to the plane, gives rise to the motion in its sense axis. 

The angle between the drive axis and the sense axis is 45°. For the sake of a preliminary analysis of the structure of the MEMS disk resonator, the simplified equations of the motion are expressed as follows [14]:

(1) $$\begin{array}{l} \ddot{x}+2\xi_{x}\omega_{x}\dot{x}+\omega_{x}^{2}x=\frac{F_{0}\sin\left(\omega_{x}t\right)}{m_{eff}}\\ \ddot{y}+2\xi_{y}\omega_{y}\dot{y}+\omega_{y}^{2}y=4A_{g}\Omega \dot{x} \end{array}$$

where *x* and *y* are the displacement of the drive axis and sense axis, *ξ* is the damping ratio, *ω* is the resonant frequency, *m_eff_* is the effective mass, *A_g_* is the angular gain, Ω is the angular rate, and F_0_ is the driving force which is defined by the input signal. 

Thus, the equation of motion in the drive axis can be expressed as

(2) $$x\left(t\right)=\frac{Q_{x}F_{0}}{k_{x}}\sin\left(\omega_{x}t-\frac{\pi }{2}\right)=x_{0}\sin\left(\omega_{x}t-\frac{\pi }{2}\right)$$

where *Q_x_* is the *Q* value of the drive mode, *Q_x_* = 1/2*ξ*_x_, *x_0_* is the amplitude of the driving axis, and *k_x_* is the stiffness of the drive axis. Assuming that the gyroscope is in mode matching, the mechanical sensitivity can be written as

(3) $$\left\{ \begin{array}{l} \left|\frac{y_{0}}{\Omega }\right|=\frac{4A_{g}Q_{x}F_{0}}{\omega_{x}m_{eff}^{2}\sqrt{{\left(\omega_{y}^{2}-\omega_{x}^{2}\right)}^{2}+{\left(\frac{\omega_{y}\omega_{x}}{Q_{y}}\right)}^{2}}}=\frac{4A_{g}Q_{x}x_{0}}{\omega_{0}}\left(k_{x}=k_{y},\omega_{x}=\omega_{y}\right)\\ m_{eff}=\underset{V}{∭}\rho \left(\varphi_{x1}^{2}+\varphi_{y1}^{2}+\varphi_{z1}^{2}\right)dV=\underset{V}{∭}\rho_{m}\left(\varphi_{x2}^{2}+\varphi_{y2}^{2}+\varphi_{z2}^{2}\right)dV\\ A_{g}=\frac{\underset{V}{∭}\rho \left(\varphi_{x1}\varphi_{y2}-\varphi_{x2}\varphi_{y1}\right)dV}{2m_{eff}} \end{array}\right.$$

where (*ϕ_x1_*, *ϕ_y1_*, *ϕ_z1_*, *ϕ_x2_*, *ϕ_y2_*, *ϕ_z2_*) are the shape functions of the disk resonator and *ρ_m_* is the density of the material.

From Equation (3), it can be concluded that the higher mechanical sensitivity can result in a larger deformation in the sense axis of the disk resonator at the same angular rate. Meanwhile, the deformation of the MEMS disk resonator in the sense axis will be transmitted to the WGM resonator and change its optic characteristics. In the next few chapters, the output value of optical detection is found to be positively correlated with the deformation of the WGM resonator. Hence, it is very important to improve the mechanical sensitivity.

Based on Equation (3), there are three possible ways to increase the mechanical sensitivity: (1) by increasing the driving force; (2) by decreasing the frequency split between the drive and sense modes through optimal design of the gyroscope; and (3) by improving the *Q* value in the drive and sense modes. 

Based on results we have previously published [14,15], the optimized parameters of the disk resonator are shown in Table 2. The frequency split and *Q* value of the resonator are 13 Hz and 90,000. 

## 3. Analysis and Simulation of the WGM Resonator

### 3.1. Operational Principle of the WGM Resonator

Another component of the gyroscope is the WGM resonator. The resonator comprised a straight waveguide and a disk optical cavity, which were placed close to each other so that light coupled from one to the other. When the circumference of a disk cavity is an integer number of wavelengths, the disk cavity is resonant to the wavelength and the light power stored in the disk builds up; this is referred to as the whispering-gallery mode (WGM). The light in WGM resonators can orbit thousands of times before escaping; therefore, the detection sensitivity of this method is expected to be greatly enhanced. The applied force on the resonator can then be detected by measuring the frequency shift of the WGM, which is caused by the physical elongation of the disk. 

Therefore, once an applied angle velocity exerts the Coriolis force on the sense axis of the MEMS disk resonator, the WGM resonator will experience the strain, which causes the resonant wavelength to shift; the angle velocity can then be determined by measuring the frequency shift of the WGM. This indicates that the WGM resonator is the key device in the sensor, and it is essential to analyze its characteristics and optimization method.

### 3.2. Approximation of the Modes in a WGM Disk Resonator

**Figure 3:**
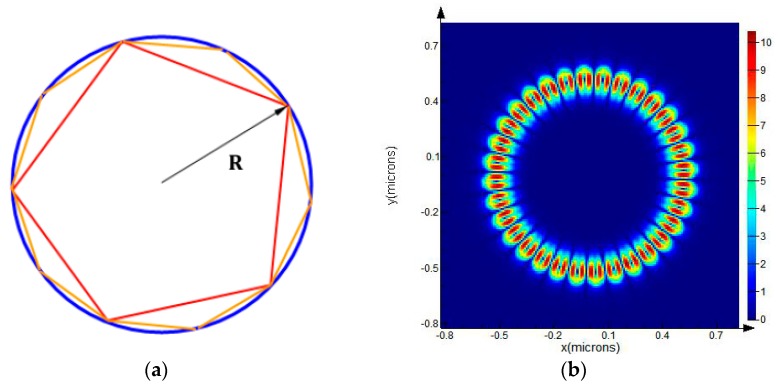
(**a**) Geometric schematic of the whispering-gallery mode (WGM) resonance; (**b**) Electromagnetic field distribution of the WGM resonance.

Considering a WGM disk resonator with a radius R, light is confined inside the resonator by total internal reflection (TIR) because the angles between the beams and the normal directions of the boundary satisfy Snell’s law. The propagation path of light inside the resonator can be approximated as a polygon, as shown in Figure 3a, and the smaller the resonance wavelength, the higher the order of the polygon. The resonators usually satisfy the condition of *R* >> *λ*_0_ so that the distance of the travelling path of photons inside the optical disk resonator is equal to its equatorial circumference [16]. Assuming that the circumference of the cavity is *L* (*L* = 2π*R*, *R* is the radius of the cavity), the resonant wavelength of the disk can be expressed as

(4) $$\lambda =\frac{n_{eff}L}{l}=\frac{2\pi Rn_{eff}}{l}$$

where *λ* is the resonant wavelength, *n_eff_* is the effective index of refraction of the optical mode in the disk cavity, and *l* is an integer representing the resonance order.

While the geometrical optics view gives an intuitive picture, a more quantitative description of the WGM is given by Maxwell’s Equations.

(5) $$\begin{array}{c} \nabla \times E=-i\omega \mu H\\ \nabla \times H=i\omega \varepsilon E \end{array}$$

In Maxwell’s equation, an isotropic medium with constant scalar permittivity and permeability is free of charge and current in order to investigate the electromagnetic fields in the disk resonators.

In the disk resonator, due to the confinement in the vertical direction, the photon is restricted in a plane. Hence, there are two polarizations: TE (E field parallel to the disk plane) and TM (E field perpendicular to the disk plane). Thus, Equation (9) becomes scalar in the *z* direction and F_z_ corresponds to H_z_ (E_z_) for the TE (TM) modes. Assuming a separable solution for F_z_(ρ,φ,z), we then have the following:

(6) $$\begin{array}{l} \Phi Z\frac{\partial^{2}\Psi }{\partial \rho^{2}}+\Phi Z\frac{1}{\rho }\frac{\partial \Psi }{\partial \rho }+\Psi Z\frac{1}{\rho^{2}}\frac{\partial^{2}\Phi }{\partial \phi^{2}}+\Psi \Phi \frac{\partial^{2}Z}{\partial z^{2}}=-\beta^{2}\Psi \Phi Z\\ \frac{1}{\Psi }\frac{\partial^{2}\Psi }{\partial \rho^{2}}+\frac{1}{\Psi }\frac{1}{\rho }\frac{\partial \Psi }{\partial \rho }+\frac{1}{\Phi }\frac{1}{\rho^{2}}\frac{\partial^{2}\Phi }{\partial \phi^{2}}+\frac{1}{Z}\frac{\partial^{2}Z}{\partial z^{2}}=-\beta^{2} \end{array}$$

If we let

(7) $$\begin{array}{l} \frac{\partial^{2}Z}{\partial z^{2}}=-\beta_{z}^{2}Z\\ \frac{\partial^{2}\Phi }{\partial \phi^{2}}=-l^{2}\Phi \\ \beta_{\rho }^{2}+\beta_{z}^{2}=\beta^{2} \end{array}$$

then we get a classic Bessel Differential Equation:

(8) $$\rho^{2}\frac{\partial^{2}\Psi }{\partial \rho^{2}}+\rho \frac{\partial \Psi }{\partial \rho }+\left[{\left(\beta_{\rho }\rho \right)}^{2}-l^{2}\right]\Psi =0.$$

In the disk resonator, the general solution of Bessel functions of the first order is as follows:

(9) $$\Psi =AJ_{l}\left(\beta_{\rho }\rho \right)+BY_{l}\left(\beta_{\rho }\rho \right).$$

*J_l_*(*β_ρ_ρ*) and *Y*_l_(*β_ρ_ρ*) represent the Bessel functions of the first and second kind, in which *l* is the angular mode number. Because *Y*_l_ (*β_ρ_ρ*) is divergent when *ρ* tends to 0, and the field should be finite at *R* = 0, the solution can be expressed as

(10) $$\begin{array}{cc} \Psi =AJ_{l}\left(\beta_{\rho }\rho \right) & \left(\rho \leq R\right) \end{array}$$

where *A* is a constant dependent on the boundary conditions.

Meanwhile, the transient field exponentially attenuates along the radial direction:

(11) $$\begin{array}{cc} \Psi =J_{l}\left(\beta_{\rho }R\right)\cdot \exp\left(-\alpha \cdot \left(\rho -R\right)\right) & \left(\rho >R\right) \end{array}$$

where $\alpha =2\pi \sqrt{n_{eff}^{2}-n_{0}^{2}}/\lambda$.

The solution of the first line in Equation (7) follows the standard slab mode calculation:

(12) $$Z\left(z\right)=\cos\left(\beta_{z}z\right)$$

where *β* = *m*π/*t*, *t* is the thickness of the disk resonator, and *m* is the longitudinal mode number.

**Figure 4:**
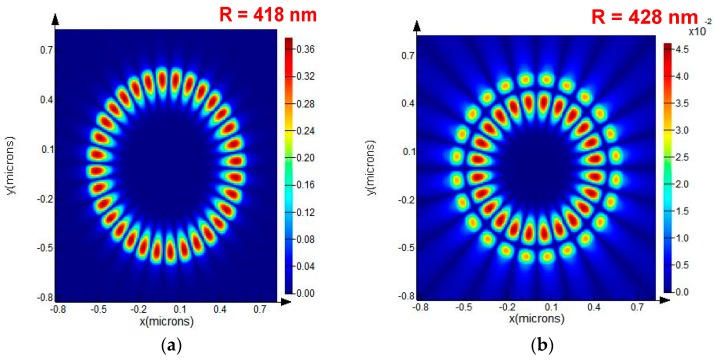
(**a**) Magnetic field of the WGM resonator in the first order (*R* = 418 nm); (**b**) Magnetic field of the WGM resonator in the second order (*R* = 428 nm).

With the help of the FDTD Solution, the first- and second-order whispering-gallery modes of the resonator were determined. Through changing the disk resonator radii from 200 nm to 600 nm in the simulation, we found that the resonator has the same resonance wavelength of 1.55 µm with radii of 418 nm and 428 nm, while the excited WGM modes are of the first and second order, respectively, as shown in Figure 4.

### 3.3. Quality Factor Q of the WGM Resonator

The loss of a resonator is an important parameter and is used to determine the sensitivity of the gyroscope. Resonator loss is commonly expressed in terms of the quality factor *Q*. The quality factor Q is related to the cavity photon lifetime *τ*, as represented in the following equation [16]:

(13) $$Q=\frac{\lambda_{0}}{\Delta \lambda }=\omega \pi$$

where *ω* is the optical frequency (*ω* = 2*πc/λ*). 

The quality factor value *Q* primarily depends on several loss mechanisms in the optical WGM resonators. The overall *Q* value can be expressed as

(14) $$Q^{-1}=Q_{couple}^{-1}+Q_{bend}^{-1}+Q_{ss}^{-1}+Q_{bulk}^{-1}+Q_{w}^{-1}$$

where *Q_couple_* is the loss induced by the coupling of the straight waveguide and the optical resonator, which will be discussed in next section; *Q_bend_* is due to the bending loss of the waveguide and the WGM resonator; *Q_ss_* is the scattering losses from the residual surface in homogeneities; *Q_w_* is the absorption losses due to contamination on the surface of the resonator; and *Q_bulk_* is the bulk absorption in the material.

### 3.4. The Model of the Theoretical Approach of Single Waveguides Coupled to WGM Resonators

**Figure 5:**
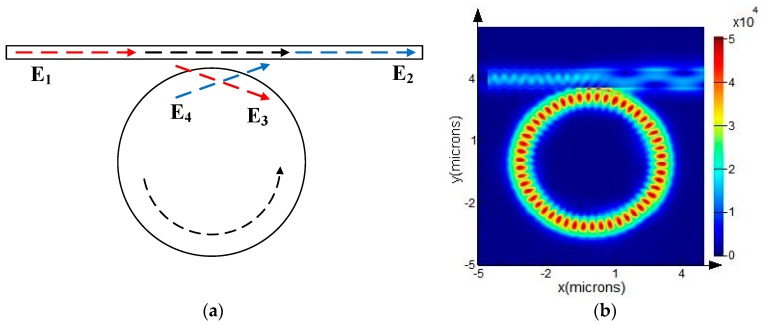
Model of the WGM resonator: (**a**) geometric optical mode; (**b**) optical field distribution.

It is impossible for a WGM resonator to perform measurement without an input–output interface. Hence, a typical resonator having a microdisk as its resonant cavity that is closely coupled with one straight bus waveguide, which serves as the input and output, was designed, as shown in Figure 5. Based on the optical field analysis performed in the previous sections, the light from the waveguide coupled to the disk was found to be an evanescent wave through the narrow gap, which is illustrated in Figure 5b.

Based on the coupled mode theory [17], the coupled mode equation is given as

(15) $$\left[\begin{array}{l} E_{2}\\ E_{3} \end{array}\right]=\left[\begin{array}{cc} t & j\kappa \\ j\kappa & t \end{array}\right]\left[\begin{array}{l} E_{1}\\ E_{4} \end{array}\right]$$

where *t* is the self-coupling coefficient of the waveguide and disk, while *κ* is the mutual coupling coefficient for both of them. *t*^2^ + *κ*^2^ = (1 − *τ*)^2^, where *τ* is the loss factor.

Solving the above equations yields the expression for the transmission factor in relation to the input waveguide:

(16) $$T={\left|\frac{E_{2}}{E_{1}}\right|}^{2}=\frac{\alpha^{2}+{\left|t\right|}^{2}-2\alpha \left|t\right|\cos\left(\theta +\phi_{t}\right)}{1+\alpha^{2}{\left|t\right|}^{2}-2\alpha \left|t\right|\cos\left(\theta +\phi_{t}\right)}$$

where *α* and *θ* represent the loss and the phase shift, respectively, after one circulation. α = e^−^*^τL^* and *θ = βL* where *β* is the propagation constant, which depends on the effective refractive index of the resonator as *β* = 2π*n*_eff_/*λ*; $\phi_{t}$ is the additional phase shift in the resonator and the length *L* = 2π*R*.

When in the resonance mode, the coupling can be considered as one of three regimes:

(1) Under-coupled regime: When the waveguide is far from the resonator, the coupling is very weak. When the waveguide moves close to the resonator, the overlap of the waveguide mode and WGMs will increase. However, when |*t*| > α, the transmission T decreases continuously from unity and approaches zero.
(2) Critical coupled regime: When the waveguide gets closer to the resonator such that |t| = α, the normalized transmission T is zero, which means that all of the input power is coupled to the resonator.
(3) Over-coupled regime: When the gap between the waveguide and the resonator is further decreased, the overlap of the modes becomes larger and the normalized transmission T becomes greater than 0 again; this is called the over-coupled regime.

**Figure 6:**
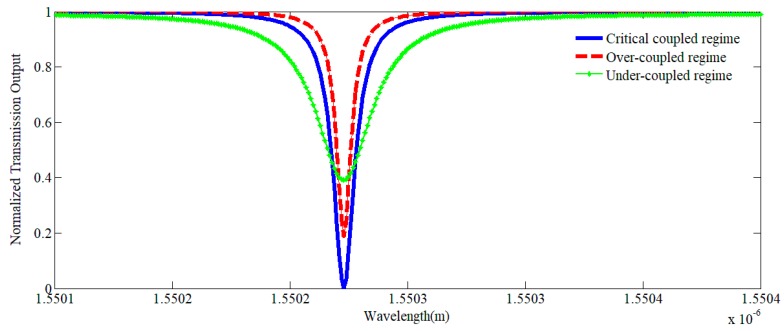
Normalized transmission output in different coupling regimes.

The classical normalized transmission spectra of the three different coupling regimes are shown in Figure 6. 

For the above scenarios, we use power normalization so that |*E*_1_|^2^ and |*E*_2_|^2^ are the respective traveling wave powers. We will, without experiencing a loss of generality, take the incident power |*E*_1_|^2^ to be unity. At resonance, $\theta +\phi_{t}=2\pi n$, *n* is an integer, and

(17) $${\left|E_{2}\right|}^{2}=\frac{{\left(\alpha -\left|t\right|\right)}^{2}}{{\left(\alpha -\alpha \left|t\right|\right)}^{2}}.$$

**Figure 7:**
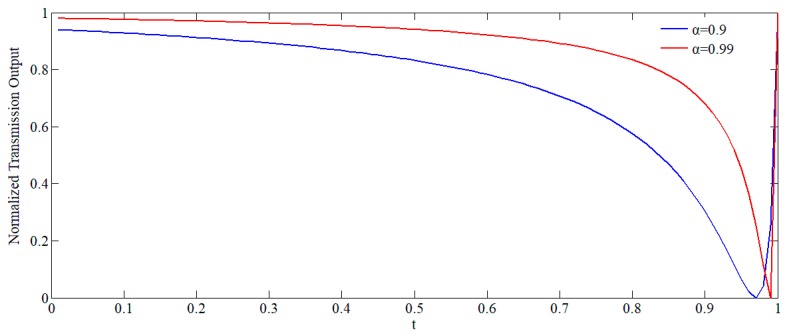
The universal transmission plot for the configuration in Figure 5.

The relationship between the light intensity and the self-coupling coefficient *t* at resonant frequency when α = 0.9 and 0.99 is plotted in Figure 7. 

For high-Q resonators, when α is close to unity, the portion of the curve to the right of the critical coupling point is extremely steep. Small changes in α for a given *t*, or vice versa, can control the transmitted power E_2_ between unity and zero. An understanding of the relationship between the deformation of the resonator caused by the rotation and α and/or *t* will allow for the identification of a method to optimize the performance of the gyroscope.

## 4. Analysis and Simulation of the MOEMS Gyroscope

In the last two chapters, the two main elements of the MOEMS gyroscope were introduced. The mechanical properties of the MEMS resonator, which converts angular velocity to displacement of the sense axis in the disk, were analyzed. At the same time, the optical properties of the WGM resonator were detailed. 

Based on that, the optional principle of the MOEMS gyroscope can be concluded. When an applied angular velocity exerts the Coriolis force on the MOEMS structure, the force on the disk resonator causes its rings to become deformed. Meanwhile, the WGM resonator will experience the same deformation for it is manufactured on the ring. The deformation of the WGM resonator also means a change in its circumference or the optical distance *L*. Based on Equation (4), the optical resonator wavelength is related to *L*. Hence, it can be found that the applied angular velocity can be measured according to the shift in the optical resonator frequency. 

In summary, the function of the MOEMS gyroscope is based on the transformation process from angular velocity to deformation of the MEMS resonator, to deformation of the WGM resonator, to a shift in the resonator frequency. In this section, the whole process will be discussed.

### 4.1. The Relationship between Frequency Shift and Deformation of the WGM Resonator

Due to physical elongation, the shape of the disk is modified. Assuming that the path length *L* of the disk is changed by Δ*L*, the shift in its resonant wavelength Δ*λ* can be expressed as follows:

(18) $$\begin{array}{l} \lambda_{m}=\frac{L_{m}n_{eff}}{l}==\frac{\left(L+\Delta L\right)n_{eff}}{l}\\ \Delta \lambda =\lambda_{m}-\lambda =\frac{L_{m}n_{eff}}{l}-\frac{L\cdot n_{eff}}{l}=\frac{\Delta L\cdot n_{eff}}{l} \end{array}$$

When the disk is strained, the diameter becomes larger in the direction of the strain and smaller in the perpendicular direction, and a circular disk becomes an ellipse. The deformation of the WGM resonator is related to the strain of the MEMS resonator for it is on the outer ring of the disk. Hence, the equation can be written as

(19) $$\Delta \lambda =\lambda_{m}-\lambda =\frac{L_{m}n_{eff}}{l}-\frac{L\cdot n_{eff}}{l}=\frac{\left\{\left[2\pi b+4\left(a-b\right)\right]-2\pi r_{0}\right\}\cdot n_{eff}}{l}$$

where *r*_0_ is the initial radius of the optical disk resonator and *a*, *b* are the semimajor axis and semiminor axis of the elliptical WGM resonator. 

Because the WGM resonator is on the MEMS resonator and its thickness is much smaller (less than 1%), the deformation of the WGM resonator is exactly the same as the strain of the silicon element below the corresponding position. Based on the above formula and the definition of strain, the following results are obtained:

(20) $$\begin{array}{l} \varepsilon =\frac{\Delta L}{L}=\frac{\left[2\pi b+4\left(a-b\right)\right]-2\pi R}{2\pi R}=\frac{\Delta \lambda }{\lambda }\\ \Delta \lambda =\varepsilon \cdot \lambda \end{array}$$

### 4.2. The Relationship between Deformation of the WGM Resonator and Stress on the MEMS Resonator 

From the last section, it is known that the resonator wavelength shift of the WGM resonator is proportional to its strain. If the relational expression between deformation of the optical resonator and stress on the disk resonator is known, the wavelength shift caused by the angular velocity can be calculated combined with Equations (3) and (20).

**Figure 8:**
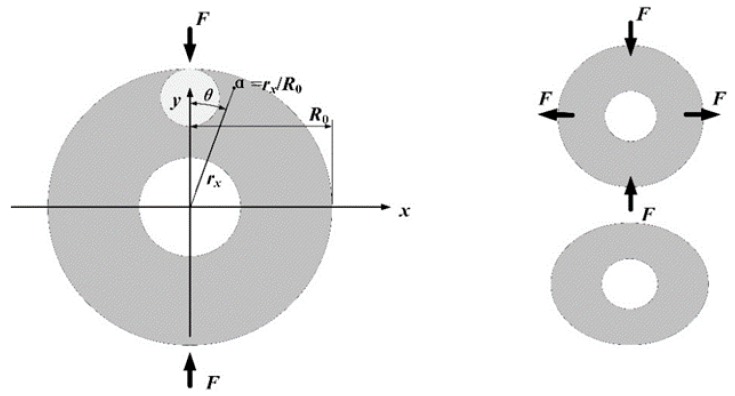
A simplified model of disk resonator deformation.

In order to facilitate the analysis, a simplified MOEMS gyro deformation model is presented, as shown in Figure 8. The radii of the optical resonators discussed in this paper are smaller than the width of the ring in the MEMS resonator, so the deformation of optical cavity is closely related to the deformation of the outermost ring caused by strain in the ring. With the help of the Timoshenko Formula, the distribution of strain in the ring can be obtained:

(21) $$\begin{array}{l} M\left(\theta \right)=\frac{1}{2}F\times R_{a}\left(\cos\theta -\frac{2}{\pi }\right)\\ \sigma_{\theta \theta }\left(\theta \right)=\pm \frac{3}{2}\frac{M\left(\theta \right)}{H\delta^{2}}=\pm \frac{3}{4}\frac{1}{H\delta^{2}}F\times R_{a}\left(\cos\theta -\frac{2}{\pi }\right) \end{array}$$

where *F* is the force in the radial direction, *R_a_* is the center diameter of the ring, *θ* is the azimuthal angle, *M*(*θ*) is the bending moment of the cross section at angle *θ*, *σ_θθ_*(*θ*) is the hoop force at angle *θ*, *H* is the thickness of the ring, and *δ* is the displacement of the ring.

Based on other researchers’ work, the complete stress distribution is [18,19]

(22) $$\begin{array}{l} \sigma_{\theta \theta }\left(\alpha ,\theta \right)=\frac{2F}{\pi R_{0}}\left[\frac{1}{2}-\frac{\left(1-\alpha \cos\theta \right)\sin^{2}\theta }{{\left(1+\alpha^{2}-2\alpha \cos\theta \right)}^{2}}-\frac{\left(1+\alpha \cos\theta \right)\sin^{2}\theta }{{\left(1+\alpha^{2}+2\alpha \cos\theta \right)}^{2}}\right]\\ \sigma_{rr}\left(\alpha ,\theta \right)=\frac{2F}{\pi R_{0}}\left[\frac{1}{2}-\frac{\left(1-\alpha \cos\theta \right){\left(\cos\theta -\alpha \right)}^{2}}{{\left(1+\alpha^{2}-2\alpha \cos\theta \right)}^{2}}-\frac{\left(1+\alpha \cos\theta \right){\left(\cos\theta +\alpha \right)}^{2}}{{\left(1+\alpha^{2}+2\alpha \cos\theta \right)}^{2}}\right]\\ \sigma_{r\theta }\left(\alpha ,\theta \right)=\frac{2F}{\pi R_{0}}\left[\frac{\left(1-\alpha \cos\theta \right)\left(\cos\theta -\alpha \right)\sin\theta }{{\left(1+\alpha^{2}-2\alpha \cos\theta \right)}^{2}}+\frac{\left(1+\alpha \cos\theta \right)\left(\cos\theta +\alpha \right)\sin\theta }{{\left(1+\alpha^{2}+2\alpha \cos\theta \right)}^{2}}\right] \end{array}$$

where *R*_0_ is the outer radius of the ring; *α* is the ratio of the distance from a point to the center to the outer radius (*α* = *R_a_*/*R*_0_); and *σ_θθ_*(*α*,*θ*), *σ_rr_*(*α*,*θ*), and *σ_rθ_*(*α*,*θ*) are the hoop force, radial force, and shear force at angle *θ* with ratio *α*, respectively.

Thus, the main strain force *τ*_max_ is

(23) $$\left\{ \begin{array}{l} p\left(\alpha ,\theta \right)=\frac{1}{2}\left[\sigma_{\theta \theta }\left(\alpha ,\theta \right)+\sigma_{rr}\left(\alpha ,\theta \right)\right]+\frac{1}{2}\sqrt{{\left[\sigma_{\theta \theta }\left(\alpha ,\theta \right)-\sigma_{rr}\left(\alpha ,\theta \right)\right]}^{2}+4\sigma_{r\theta }^{2}\left(\alpha ,\theta \right)}\\ q\left(\alpha ,\theta \right)=\frac{1}{2}\left[\sigma_{\theta \theta }\left(\alpha ,\theta \right)+\sigma_{rr}\left(\alpha ,\theta \right)\right]-\frac{1}{2}\sqrt{{\left[\sigma_{\theta \theta }\left(\alpha ,\theta \right)-\sigma_{rr}\left(\alpha ,\theta \right)\right]}^{2}+4\sigma_{r\theta }^{2}\left(\alpha ,\theta \right)}\\ \tau_{\max}\left(\alpha ,\theta \right)=\frac{p-q}{2}=\frac{1}{2}\sqrt{{\left[\sigma_{\theta \theta }\left(\alpha ,\theta \right)-\sigma_{rr}\left(\alpha ,\theta \right)\right]}^{2}+4\sigma_{r\theta }^{2}\left(\alpha ,\theta \right)} \end{array}\right.$$

The strain ε can be calculated by

(24) $$\varepsilon =\frac{\sigma }{E}=\frac{\iint_{S}f\left[\alpha ,\theta ,\tau_{\max}\left(\alpha ,\theta \right)\right]d\delta }{E}$$

where *E* is the Young’s modulus of the ring, *σ* is the combined stress on the element corresponding to the location of the WGM resonator, and *f*(*α*,*θ*,*τ*) is the surface equation of the strain force. The ring coordinates (*α*,*θ*) are transformed from the orthogonal coordinates (*x*,*y*):

(25) $$\left\{ \begin{array}{l} \alpha =\frac{\sqrt{x^{2}+y^{2}}}{R_{0}}\\ \theta =\arctan\frac{x}{y} \end{array}\right.$$

**Figure 9:**
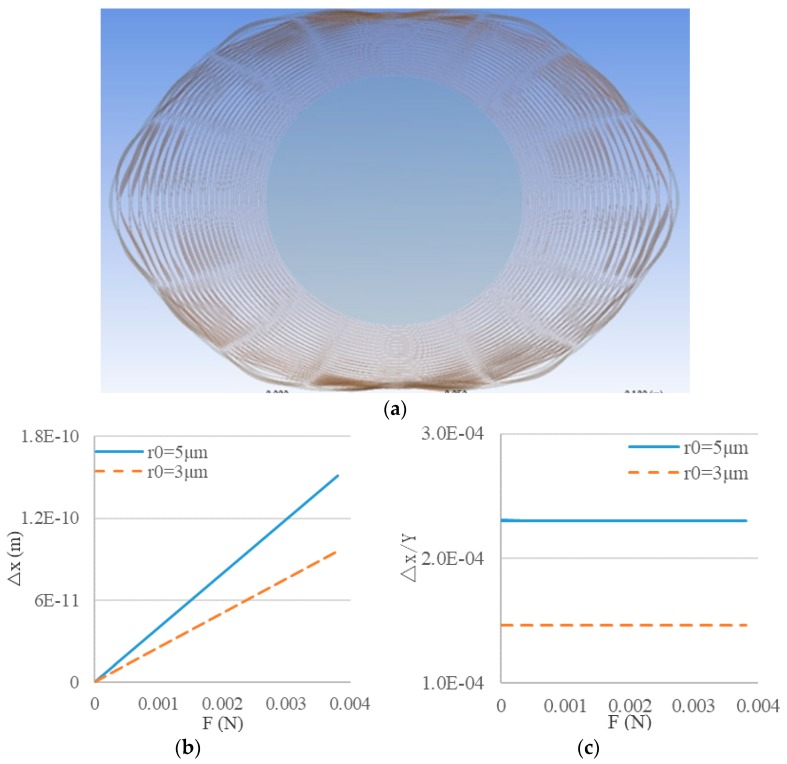
(**a**) Deformation of the MEMS disk resonator under static force; (**b**) Radius variation of the optical resonator; (**c**) Ratios of radii variation in optical and MEMS resonators.

Considering the complexity of the disk resonator, it is impossible to get the analytic solutions directly, so the relationships between deformation of the disk resonator and the optical cavity with different radii were analyzed with the help of the finite element simulation software ANSYS Workbench. The results of the static simulation are shown in Figure 9.

Using the simulation results, more credible results were obtained in support of the theoretical analysis above:

(26) $$\left\{ \begin{array}{l} \Delta x=K_{Yx}Y\\ \Delta y=K_{Yy}Y \end{array}\right.$$

where △*x* and △*y* are the variations of the WGM resonator radius in the tangential and radial directions of the disk resonator, respectively; *Y* is the maximum displacement of the disk resonator in the force direction; and *K_Yx_* and *K_Yy_* are coefficients concluded from the simulation results. When *r*_0_ = 5 µm or 3 µm, *K_Yx_* = 2.301 × 10^−4^ or 1.4616 × 10^−4^ and *K_Yx_* = 3.807 × 10^−6^ or 2.438 × 10^−6^.

## 5. Simulation of the MOEMS Gyroscope

In the last section, the transformation processes of deformation to a resonant wavelength shift in the WGM resonator and stress on the MEMS resonator to deformation of the WGM resonator were discussed. In this section, a simulation test was used to prove the working principle of the MOEMS gyroscope and calculate its sensitivity and cross-axis sensitivity.

### 5.1. Simulation Analysis of the Resonant Wavelength Shift of a Deformed WGM Resonator

Based on the conclusion of Section 4, the semimajor axis and semiminor axis of the elliptical WGM resonator are *r_x_* = *R* ± △*x* and *r_y_* = *R* ± △*y*. Because △*x* >> △*y*, the variation △y can be ignored, and the maximum of wavelength shift is

(27) $$\frac{\Delta \lambda_{\max}}{\lambda }=\frac{\Delta L}{L}\approx \frac{\left[2\pi b+4\left(a-b\right)\right]-2\pi R}{2\pi R}\approx \frac{\pm 4\Delta x}{2\pi r_{0}}=\frac{\pm 2\Delta x}{\pi r_{0}}=\frac{\pm k_{x\lambda }\Delta x}{r_{0}}.$$

**Figure 10:**
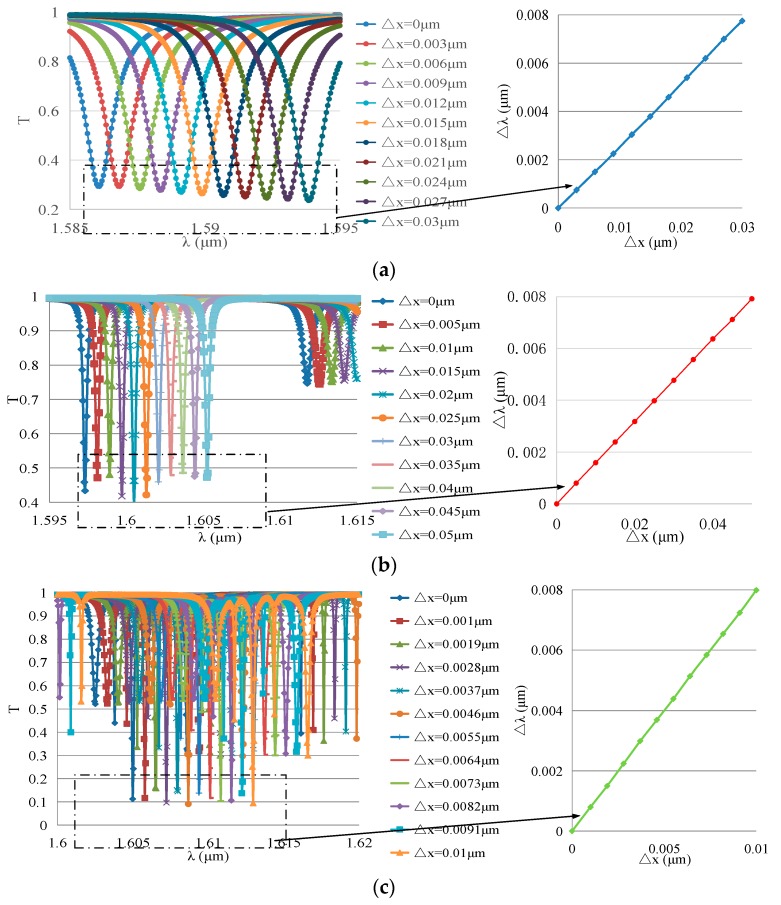
FDTD simulation results of the relationship between △x and △λ in WGM resonators with different radii: (**a**) *r*_0_ = 3 µm; (**b**) *r*_0_ = 5 µm; (**c**) *r*_0_ = 10 µm.

Assume that *r*_0_ = 3 µm, 5 µm, or 10 µm and that △*x* = (10^−4^ ~ 0.01)*r_0_; with the help of FDTD Solutions, the transmission spectrum of the WGM resonators can be calculated. With △*x* as the transverse axis and △*λ* as the longitudinal axis, the results are arranged as shown in Figure 10. 

According to the curves on the right-hand side in Figure 10a–c, the resonant wavelength shift △*λ* is proportional to the deformation △*x* in WGM resonators with different radii.

**Figure 11:**
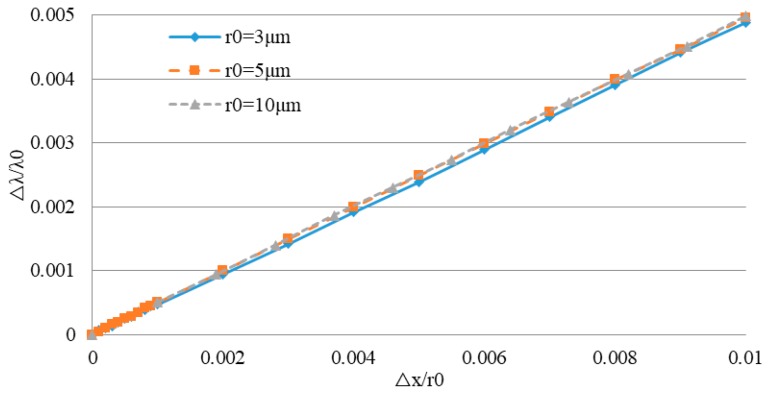
Relationship between △*x*/*r*_0_ and △*λ*/*λ*_0_ in WGM resonators with different initial radii.

Dividing the deformation △*x* by the initial radius *r*_0_ and comparing this with the resonance wavelength shift △*λ* divided by the initial resonance wavelength *λ*_0_ gives the results shown in Figure 11.

Comparing this with Equation (27), it can be seen that the relative variation of the resonance wavelength λ/λ_0_ is proportional to the strain *x*/*r*_0_, and the scale factor *k_xλ_* is independent of the initial radius ($\frac{\Delta \lambda }{\lambda }=k_{x\lambda }\frac{\Delta x}{r_{0}}$, *k_xλ_* ≈ 0.4486). Hence, the results obtained by simulation and by theoretical calculation are similar. 

### 5.2. Key Specifications of the MOEMS Gyroscope 

In Section 4.2, the relationship between deformations of the MEMS resonator and the optical resonator was preliminarily concluded from static structure simulations. With the help of harmonic response simulation, the WGM resonator deformation at an applied angular velocity can be calculated. By combining all the conclusions above, some key specifications of the gyroscope were estimated.

**(1) Sensitivity**

**Figure 12:**
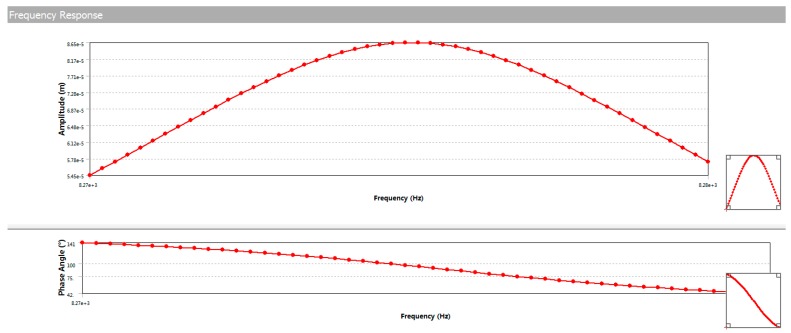
Harmonic response simulation results of the MEMS resonator.

Assuming that the drive force of the gyroscope is 4.6 × 10^−6^ N, the resulting structural parameters of the MEMS resonator are given in Table 2; the resonant frequencies of the drive and sense modes are 8324.8 Hz and 8326.2 Hz, and the results of the harmonic response simulation are shown in Figure 12. It can be seen that the maximum drive amplitude of the MEMS resonator was 2.9329 µm (*X*_0_ = 2.9329 µm) when it was at the resonance point.

Combining Equations (3), (26), and (27), we get

(28) $$\left\{ \begin{array}{l} \left|\frac{Y}{\Omega }\right|=\frac{4A_{g}Q_{x}X_{0}}{\omega_{0}}\\ \Delta x=K_{Yx}Y\\ \frac{\Delta \lambda_{\max}}{\lambda }=\frac{\Delta L}{L}\approx \frac{\pm k_{x\lambda }\Delta x}{r_{0}} \end{array}\right.$$

Hence, the sensitivity of the MOEMS gyroscope is

(29) $$S_{MOEMS}=\left|\frac{\Delta \lambda }{\Omega }\right|=\left|\frac{\Delta L}{\Omega L}\lambda \right|\approx \left|\frac{\left[2\pi b+4\left(a-b\right)\right]-2\pi R}{2\pi R\Omega }\lambda \right|=\frac{k_{x\lambda }K_{Yx}}{r_{0}}\frac{4A_{g}Qx_{0}}{\omega_{0}}\lambda$$

Considering the Q value and the complexity of the transmission spectrum, a WGM resonator with a 5 µm radius was selected in the MOEMS gyroscope; consequently, *λ*_0_ = 1.59732 µm, *K_yx_* = 2.301 × 10^−4^, and *K_xλ_*= 0.4886. Substituting all these parameters into Equation (29), we get S_MOEMS_ = 3.002 pm/(°·s^−1^).

**(2) Measurement Range**

**Figure 13:**
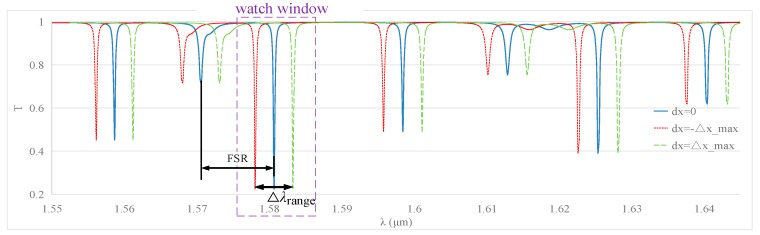
Transmission spectra drift of the MOEMS gyroscope.

Once an angular velocity is applied to the MOEMS gyroscope, its transmission spectra will drift due to the resonance wavelength shift. In order to avoid inaccuracies, the drift (△λ_range_) should be less than the separation between successive resonances; this separation is also called the free spectral range (FSR). Meanwhile, for the sake of minimizing the interference from transmission spectra overlapping, we set △*λ*_range_ = 0.5*FSR. Based on the results of the FDTD simulation, as shown in Figure 13, it can be seen that FSR = 0.01004 µm and △*λ*_range_ = 5.02 nm. If the watch window is set in a proper range, the drifting is clear and the shift in the resonance wavelength can be picked up easily.

Knowing △λ_range_, the range of measurable angle velocity can be calculated by $\Omega_{range}=\pm \frac{1}{2}\cdot \left|\frac{\Delta \lambda_{range}}{S_{MOEMS}}\right|=\pm 836.11{}^{\circ}/s$.

**(3) Nonlinear error**

**Figure 14:**
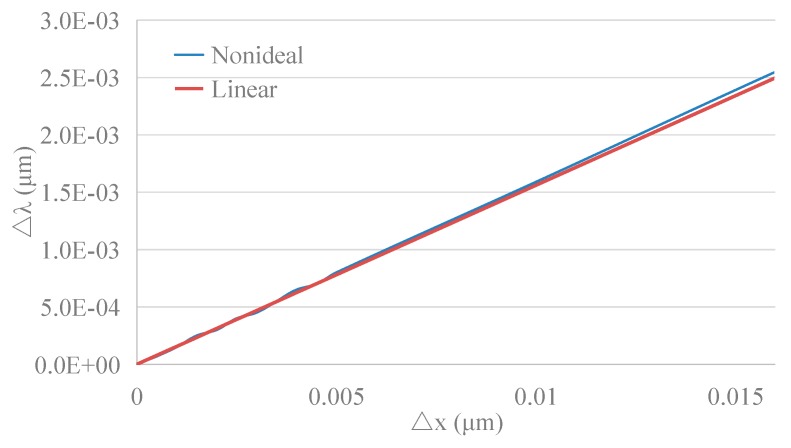
The output of the MOEMS gyroscope in linear and nonideal conditions.

Within the measurement range of the MOEMS gyroscope, the maximum shift in the resonance wavelength is ±2.51 nm; based on Equation (27), the radius variation of the WGM resonator, △x, is about ±16.4679 nm. Hence, by comparing the linear and the nonideal results, as shown in Figure 14, the nonlinear error is calculated by following equation.

(30) $$\delta =\frac{d{\left(\Delta \lambda \right)}_{\max}}{\Delta \lambda_{range}/2}\times 100\%$$

Thus, the nonlinear error of the MOEMS gyroscope in measurement is about 1.67% based on the simulation results.

**(4) Cross-Axis Coupling and Equivalent Signals**

In the gyroscope, due to fabrication imperfections disrupting the isotropy of the MEMS disk resonator, causing frequency mismatch and mode coupling [20], we consider the cross-axis coupling. Equation (1) can be re-written as

(31) $$\begin{array}{l} m_{eff}\ddot{x}+2m_{eff}\xi_{x}\omega_{x}\dot{x}+\omega_{x}^{2}m_{eff}x+k_{yx}y=F_{0}\sin\left(\omega_{x}t\right)\\ \ddot{y}+2\xi_{y}\omega_{y}\dot{y}+\omega_{y}^{2}y=k_{xy}x+4A_{g}\Omega \dot{x} \end{array}$$

where k_xy_ and k_yx_ are the equivalent cross-axis-coupling stiffness.

**Figure 15:**
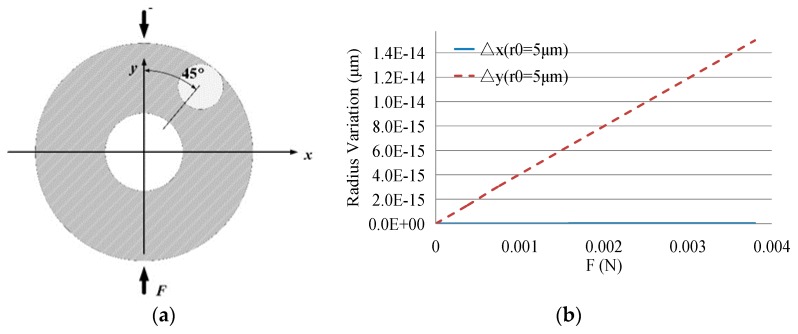
(**a**) A simplified model about cross-axis coupling in disk resonator. (**b**) The comparison of cross-axis coupling signal and drive signal.

According to the method in Section 4.2, the simulation was used to analyze the cross-axis coupling. The position of the WGM resonator and applied force is shown in Figure 15a, while the results of the optical resonator deformation under variable force are given in Figure 15b.

Thus, we can get the relationship between the deformations of the two elements:

(32) $$\left\{ \begin{array}{l} \Delta x\approx 0\\ \Delta y=K_{Xy,q}X \end{array}\right.$$

where *K_Xy,q_* is the coefficient concluded from the simulation results. When *r*_0_ = 5 µm, *K_Yx_* = 2.28 × 10^−8^.

Referring to Equation (33), the cross-axis coupling can be calculated as follows.

(33) $$\left\{ \begin{array}{l} \Delta y=K_{Xy,q}X_{0}\\ \Delta \lambda_{q}=\left|\frac{\Delta L}{L}\lambda \right|\approx \frac{k_{x\lambda }\Delta y}{r_{0}}\lambda \end{array}\right.$$

Thus, the wavelength shift caused by cross-axis coupling is about 0.01 pm. Based on the sensitivity of the gyroscope, the orthogonal signal which is equivalent to the angular velocity is about 0.01°/s when only the cross-axis coupling is considered.

## 6. Process and Result of Fabrication

**Figure 16:**
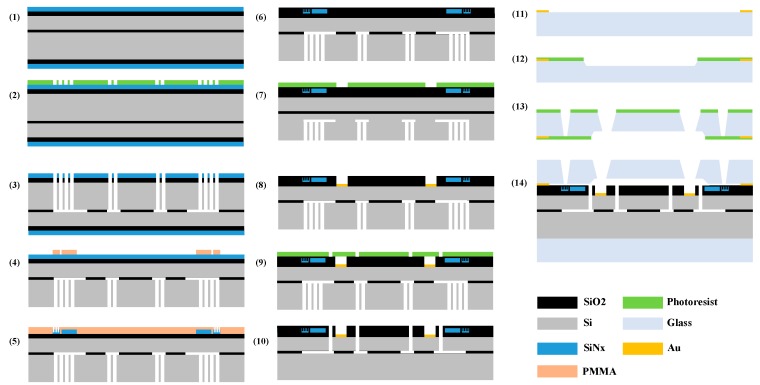
The fabrication process of the MOEMS gyroscope.

The gyroscope can be fabricated in a special silicon-on-insulator (SOI) wafer with five layers. From the top to the bottom of the wafer is an SiO_2_ layer 2 µm thick, a device layer (Si) 60 µm thick, a buried oxide layer 1µm thick, a handle layer 400 µm thick, and an SiO_2_ layer 2 µm thick. The device layer and the handle layer are made on low-resistivity, boron-doped (P-type) <100> single crystal silicon wafer. BF33 borosilicate glass was chosen as the material for the substrates and for the cap of the gyroscope due to its close coefficient of thermal expansion to that of silicon. The main process flows are shown in Figure 16:

(1) A 2 µm plasma-enhanced chemical vapor deposition (PECVD) silicon nitride (SiN_x_) layer was deposited on the top and bottom sides of the SOI wafer.
(2) The photoresist was put on the bottom side of the wafer through spin coating and patterned by mask1 to define the holes in the handle layer.
(3) The through-holes in the handle layer were formed through deep reactive ion etching (DRIE), and then part of the buried oxide layer was etched by the input KOH vapor from the holes.
(4) The bottom SiO_2_ layer and SiN_x_ layer were polished after Step (3), then a layer of PMMA was applied on the top SiN_x_ layer. After that, the pattern of the optical disk resonator and the wave guide was defined using e-beam lithography.
(5) The remaining PMMA was washed off after the WGM resonator and the wave guide were created through reactive ion etching (RIE), and then a new PMMA layer was applied on the same side. Next, the pattern of the grating coupler was defined on the PMMA using e-beam lithography, and the coupler was made with the help of inductively coupled plasma (ICP) etching.
(6) The PMMA was cleaned off after Step (5), and then another SiO_2_ layer was deposited on the front of the wafer to protect the optical devices made in the last step.
(7) The photoresist was spun on the deposited SiO_2_ layer, and then the pattern of metal pads was defined on the photoresist using mask2.
(8) With the pattern from Step (7), the position of the pad in the device layer was exposed after wet etching, and then 30/300 nm thick Cr/Au was deposited in the pattern to form the pads through a lift-off process. After that, the remaining photoresist was cleaned off the wafer.
(9) We spun the photoresist again and defined the pattern of the MEMS disk resonator in the photoresist using mask3.
(10) The resonator structure and the electrodes were simultaneously released using the Bosch ICP.
(11) The pattern of the bonding point and the metal envelope wall in the glass, which would be used for gold silicon bonding, was created. As in Steps (7) and (8), the pattern was defined using mask4 and the object was formed through a lift-off process.
(12) We spun the photoresist on the glass and then defined the pattern of the cavity using mask5, then wet etched the cavity in the glass.
(13) We spun the photoresist on another side of the glass and defined the pattern of the through holes using mask6, then wet etched the holes in the glass.
(14) The glass cap was bonded with the SOI through gold–silicon bonding with the bonding point and the metal envelope wall. Then, another glass wafer was bonded on the bottom of the SOI wafer using the anodic bonding method.

## 7. Conclusions

The results show that microdisk WGM resonators will experience optical resonance shifts when exposed to the deformation force caused by an MEMS resonator under an external angular velocity. The analysis and simulation results explained the operational principle of the optical gyroscope and gave us a few different ways to improve the performance of the gyroscope through mechanical and optical elements. With these results, some key specifications including sensitivity, measurement range, nonlinear error, and bias instability were calculated and analyzed. The fabrication process was also discussed. These results are only the first step towards the design and development of angular velocity sensors that are based on this principle; our future work will focus on manufacturing, experiments, and improvement of the sensor. 

## Figures and Tables

**Table 1:** Information about each layer in Figure 1e.

No.	Name	Material	Thickness(µm)
e-1	Gap layer	Glass	100
e-2	Sealing layer	Au/Cr/Sn	1
e-3	Optical components layer	SiN_x_	0.35
e-4	Metal pads layer	Au/Cr	0.3
e-5	Optical substrate layer	SiO_2_	2
e-6	Device layer	Doped silicon	60
e-7	Buried oxide layer	SiO_2_	2
e-8	Handle layer	Monocrystalline silicon	500

**Table 2:** The parameters of the selected MEMS disk resonator.

Parameter	Value
Spoke number	16
Ring number	60
Spoke width	20 µm
Spoke length	10 µm
Ring width	20 µm
Electrode gap	1.5 µm
